# Optimizing an Adolescent Hybrid Telemedical Mental Health Service Through Staff Scheduling Using Mathematical Programming: Model Development Study

**DOI:** 10.2196/43222

**Published:** 2023-03-28

**Authors:** Abigail Palmer, Gemma Johns, Alka Ahuja, Daniel Gartner

**Affiliations:** 1 School of Mathematics Cardiff University Cardiff United Kingdom; 2 Aneurin Bevan University Health Board National Health Service Newport United Kingdom

**Keywords:** linear Programming, telemedicine, remote consultation, mental health, teen, adolescent, mental disorder, disorder, disease, youth, decision, support, tool, model

## Abstract

**Background:**

According to the World Health Organization, globally, one in seven 10- to 19-year-olds experiences a mental disorder, accounting for 13% of the global burden of disease in this age group. Half of all mental illnesses begin by the age of 14 years and some teenagers with severe presentations must be admitted to the hospital and assessed by highly skilled mental health care practitioners. Digital telehealth solutions can be useful for the assessment of young individuals remotely. Ultimately, this technology can save travel costs for the health service rather than assessing adolescents in person at the corresponding hospital. Especially in rural regions, where travel times can be high, this innovative approach can make a difference to patients by providing quicker assessments.

**Objective:**

The aim of this study is to share insights on how we developed a decision support tool to assign staff to days and locations where adolescent mental health patients are assessed face to face. Where possible, patients are seen through video consultation. The model not only seeks to reduce travel times and consequently carbon emissions but also can be used to find a minimum number of staff to run the service.

**Methods:**

To model the problem, we used integer linear programming, a technique that is used in mathematical modeling. The model features 2 objectives: first, we aim to find a minimum coverage of staff to provide the service and second, to reduce travel time. The constraints that are formulated algebraically are used to ensure the feasibility of the schedule. The model is implemented using an open-source solver backend.

**Results:**

In our case study, we focus on real-world demand coming from different hospital sites in the UK National Health Service (NHS). We incorporate our model into a decision support tool and solve a realistic test instance. Our results reveal that the tool is not only capable of solving this problem efficiently but also shows the benefits of using mathematical modeling in health services.

**Conclusions:**

Our approach can be used by NHS managers to better match capacity and location-dependent demands within an increasing need for hybrid telemedical services, and the aims to reduce traveling and the carbon footprint within health care organizations.

## Introduction

### Overview

Worldwide, 10%-20% of children and adolescents experience mental health disorders, and delivering appropriate and timely child and adolescent mental health care challenges health services [1]. These challenges are often reported as structural, such as cost, transportation, or time constraints [2]. Alternative approaches to delivering mental health services in a way that is safe, appropriate, and timely can reduce cost and reduce the time to, for example, assess patients [2-7].

The use of technology to deliver health care is a growing area of clinical practice and research interest [2]. This approach involves using interactive technology to allow professionals, patients, and families at different geographic locations to interact as if they were meeting in person [2,3]. Using technology in this way is referred to as “telehealth” [8] and is being used globally and widely to deliver health care via assessment, consultation, and treatment comparable to in-person care [9-13]. Overall, the COVID-19 pandemic appears to have been a positive transition to the use of telepsychiatry, with it being viewed as a convenient and safe way to provide patient care [14,15].

Telehealth is reported to be beneficial for mental health provision and is becoming more widely adopted with children and adolescents, with mental health reported to be among the most active pediatric specialties [16-20]. Telehealth offers an opportunity to bridge the gap between supply and demand in child and adolescent mental health services [4]. There is a consensus of acceptability and satisfaction [21]. It can offer access to care that may have otherwise been unavailable, particularly in rural and other underserved settings [4]. It is reported to have improved resource eﬀiciency and increased cost and time savings compared to traditional modes of practice [22]. It can allow health professionals to extend boundaries of practice and overcome barriers of proximity [22], increasing their daily scope and ability to work and extending their reach and integration of expertise into other services [23].

In the United Kingdom, the delivery of child and adolescent mental health services via telehealth is relatively new, compared to other countries such as Canada, Australia, and New Zealand, where it is widely used. Several pilot studies have been conducted in recent years in the United Kingdom; however, the sustainability of these projects tends to be the failure of health care staff in continuing its use after the pilot phase. This tends to be associated with factors such as busy staff naturally returning to their normal work patterns.

In this study, we developed a mathematical program that schedules mental health care practitioners and assigns them to either the base of the telehealth care service or to hospitals where staff would be required to assess or treat patients. Mathematical programming is a part of constrained optimization, and Crown et al [24] have provided a comprehensive definition and illustrations of the methodology in health care. We used this paradigm to find a minimum coverage for staff by satisfying time- and location-dependent demands and to minimize travel distances.

### Background

In 2019, The Health Foundation [25] funded a project within the Aneurin Bevan University Health Board, a National Health Service (NHS) Trust in Wales, for 15 months to establish a telehealth innovation for an improvement program with the objective to provide mental health appointments or assessments to children and adolescents. This newly established program is called “Connecting with Telehealth to Children in Hospital and Healthcare” (CWTCH). It is a quality improvement NHS project offering safer and more eﬀicient appointments to the Child and Adolescent Mental Health Service (CAMHS) using telehealth technology. The aim of this project is to pilot a telehealth project in a CAMHS setting and to evaluate the use, suitability, and satisfaction among patients, parents, and professionals across a range of settings to demonstrate improved access and reduced waiting times to CAMHS, and time and cost savings for patients, their parents, and staff.

### Introducing a Hub and Spoke Model

The telehealth technology is set up and linked to a range of health care and education sites. There is a main hub site (the current CAMHS site), which is in private rooms. The CAMHS staff involved in the CWTCH project included specialist CAMHS consultant psychiatrists, emergency liaison teams, and specialist eating disorder teams. The CAMHS hub site provides a wide range of telehealth services to other health care sites (which act as remote yet transferable spokes, in that the technology can be carried from one site to another by the research team using a highly secure and password-protected iPad or laptop device). These sites include, but are not limited to, conducting emergency assessments in pediatric wards, medicine reviews and follow-up appointments delivered at home, and early diagnosis and intervention delivered to general practices and school settings.

A referral to the hub site can be made by any member of staff working within the designated spoke sites. Initially, the spoke sites would contact the CAMHS team and request an appointment, and, if available, a time slot would be scheduled (often on the same day), and additional information about the project and consent forms would be electronically sent to the spoke site or delivered in person by the research team. A web link to the telehealth application (Attend Anywhere) would be sent to the person arranging the referral. This link would request the name, date of birth, and contact telephone number (in case of technology failing or disconnection) of the person who is having the appointment (eg, school pupil, patient, or parent). Once the site is connected, the hub site would be alerted that the patient is waiting in the “CAMHS Virtual Waiting Room.” The CAMHS member of staff would then join the call and start the appointment.

### The Equipment

At the CAMHS site, there are a range of fully equipped private rooms that are set up with the technology required for taking appointments. This includes a PC at the hub site and an iPad or laptop device for the sites, all of which have a webcam, microphone, and speaker incorporated. As this is a quality improvement project set within the NHS, there are strict guidelines and regulations to follow when providing clinical care and outputs; therefore, a specialized communication platform has been used on all equipment used for the CWTCH project. This package is a safe and secure version of picture-to-picture style video (ie, the video is linked to both sites). The clinician end of the platform is password-protected and only accessible to a professional working within the hub site and is signed up to be part of the CWTCH project. The site is only available via a safe and secure link that is sent on to the user as a referral. The objective of our model is, given all the staﬀing and equipment constraints, how can we provide an eﬀicient service that minimizes the number of staff required and contains the miles traveled to the different hospital sites.

## Methods

In what follows, we provide a formulation of the mathematical model. We start with the sets and indices for modeling staff and locations. Furthermore, we turn to model parameters such as demand. Afterward, we will introduce the decision variables, model objective, and constraints.

### Sets, Indices, and Parameters of Our Mathematical Model

Let 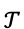 denote the planning horizon, and let 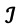 be the set of staff. Let 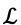 denote the set of locations where staff are assigned to work. Let *d_l,t_* denote the expected demand at location *l* ∈ 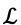 on day *t* ∈ 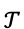. Let *w_i_* denote the number of patients that staff member *i* can treat. Furthermore, let *m_i,l_* denote the miles traveled (back and forth) if staff member *i* must travel to location *l* to see patients, which includes the miles to drive back to the home base. Table 1 provides an example that can be set up in a spreadsheet.

**Table 1:** Parameter setting for the distance parameter m_i,l_.

Name	Hospital 1 (miles)	Hospital 2 (miles)	Hospital 3 (miles)
Kelly	0	20	30
James	44	25	0
Olivia	20	0	30
Amelia	28	17	0
Joseph	0	21	38
Emily	32	0	18
Matthew	29	0	35
Laura	0	24	36

### Decision Variables

We introduce the binary decision variables *x_i,l,t_* = 1 if staff member *i* ∈ *I* works at location *l* ∈ *L* on day *t* ∈ *T*, 0 otherwise.

### Objective Function

Our objective is to find a minimum staﬀing pattern to run the hybrid telehealth CAMHS service. Accordingly, we have the following objective function:

minimize *z* = Σ*_i_* Σ*_l_* Σ*_t_ x_i,l,t_*                                       **(1)**

To reduce travel costs, the CAMHS service decided to evaluate a second objective, which can be described mathematically using objective function (2). It minimizes the distance travelled by staff.

minimize z = Σ*_i_* Σ*_l_* Σ*_t_*
*m_i,l_* ⋅ *x_i,l,t_*                            **(2)**

### Constraints

Because we want to cover the demand, the demand satisfaction constraint is as follows:




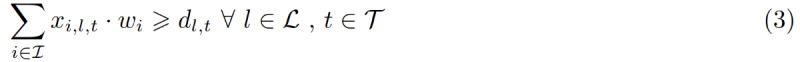




A member of staff cannot be assigned to multiple locations at the same time. Accordingly,




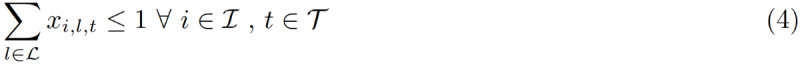




Finally, we introduce the decision variables and domains, which are represented using equation (5).




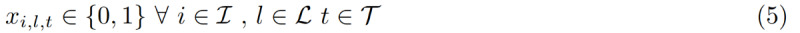




### Ethical Considerations

This study was a service improvement study and was exempt from institutional review board approval. Study data were anonymized and deidentified.

## Results

We implemented the mathematical model in a decision support tool based on Excel (Microsoft Corp) using Open Solver and the COIN-OR optimization library.

### Modeling the Weekday and Site-Dependent Demand

We used information from 2015 to 2018 to predict the demand at the different hospital sites. Table 2 provides a summary statistic about the weekday-dependent demand distribution.

The numbers reveal that on weekends, there is substantially lower demand than that on weekdays. Another observation is that more patients are seen at the beginning of the week. To predict site and weekday-dependent demand, we tested different time-series models such as additive and multiplicative decomposition approaches, exponential smoothing, and the Holt-Winters model, but the best performance was achieved using a multiple regression model with day of the week, bank holiday, and month as independent variables. We then linked the predictions from the multiple regression model with the mathematical program introduced in the previous section. This is implemented in Excel using an open-source solver, as the next sections will reveal.

**Table 2:** Weekday-dependent demand distribution.

	Monday	Tuesday	Wednesday	Thursday	Friday	Saturday	Sunday
Mean	2.42	2.65	2.23	2.16	2.20	1.29	1.43
Median	2	3	2	2	2	0	1
Mode	1	1	1	1	1	0	1
Minimum	0	0	0	1	1	0	0
Maximum	8	7	6	5	5	3	3
Range	8	7	6	4	4	3	3
Total	109	106	98	97	99	31	40

### Setting Up Staff Availability

Mental health care practitioners are either full-time, in which case they work 5 days a week and have 2 days off, or part-time, which varies from 2 to 4 working days in a week. In the interest of fairness, each practitioner works 2 weekends a month, as we expect there to be a demand 7 days a week. We have also taken into consideration that full-time workers will take approximately 3 days’ leave a month and part-time workers take 1-2 days’ leave depending on their hours.

### Implementation in Excel

**Figure 1:**
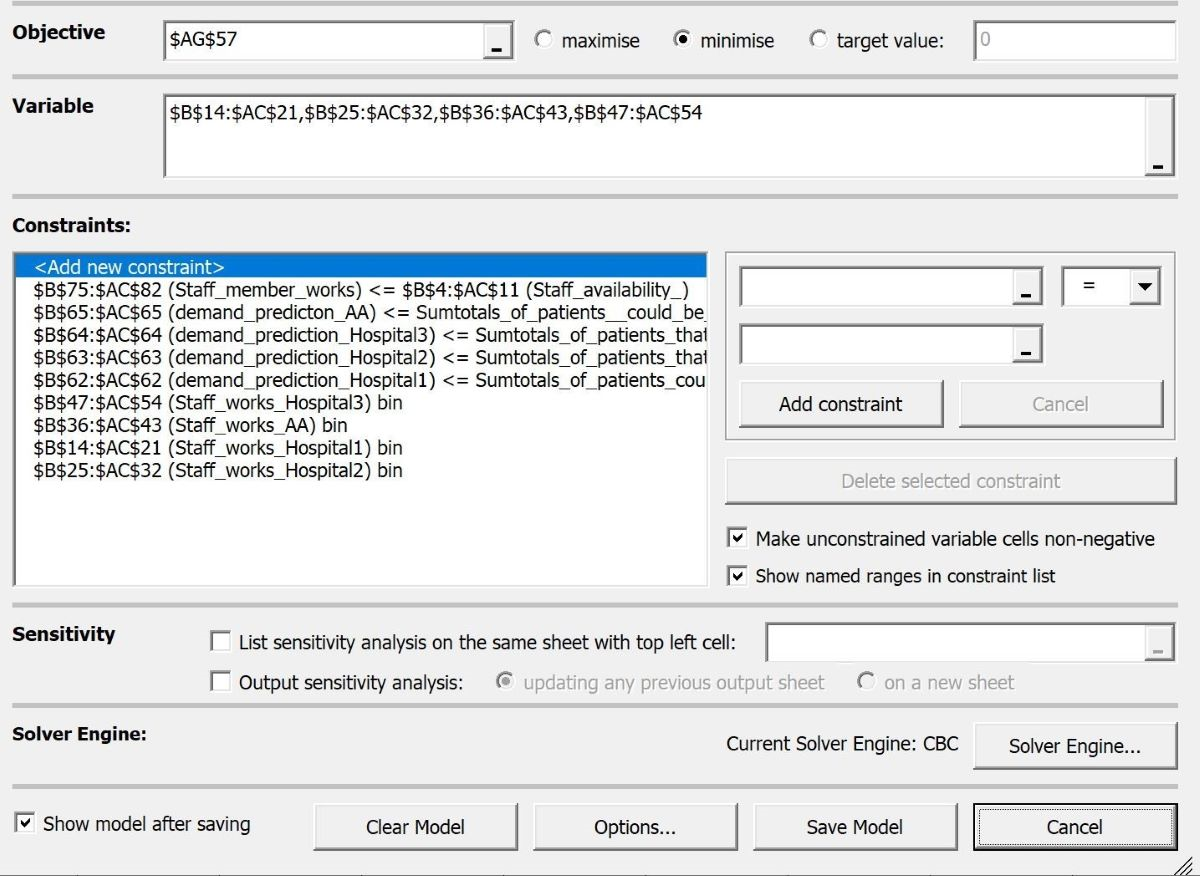
Objective function and constraints parametrized in OpenSolver.

The model is made up of binary variables as a member of staff is either working (=1) or not (=0). The model must fit into the constraints as a mental health care practitioner cannot be sent to 2 separate locations at the same time. We also must ensure that there are enough staff to cover the demand of each hospital. Named ranges have been added to the model to further clarify what each constraint represents. The hospital names have been generalized to hospitals 1, 2, and 3, while “AA” stands for the telehealth application Attend Anywhere. Figure 1 shows how the mathematical model is implemented in OpenSolver.

### Solution

As our mathematical model is 3D, we had to create separate matrices for each of the distinct locations. We created hidden spreadsheets with 0-1 matrices, which represent the site-dependent rota. OpenSolver then reads these matrices and creates the constraints of our model in the backend. To increase the usability of the tool, we translated these matrices into the format shown in Tables 2 and 3 for the rota and mileage, respectively.

In what follows, we will provide the results of solving the models with the 2 different objectives. Table 3 provides the solution for a 1-week rota and objective function (1). Staff are scheduled 31 times, which means that they are off duty 21 times. The total number of miles travelled is 416. For simplicity, Tables 2 and 3 show 1-week rota and miles, but OpenSolver is set up to solve a 4-week problem (see Table 4).

Table 5 shows the mileage for the second objective; that is, minimizing travel distance for the staff. Although the staff are scheduled for the same number of days (n=31), only 47 miles are travelled in that week. One explanation of this phenomenon is that more mental health care practitioners are allocated to sites closer to their base; hence, by solving via the second objective with our current input values, we can save 369 miles being travelled by them while still using the same number of staff.

**Table 3:** Solution with the first objective.

	October 14, 2019	October 15, 2019	October 16, 2019	October 17, 2019	October 18, 2019	October 19, 2019	October 20, 2019
Kelly	Hospital 1	Hospital 3	AA^a^	OFF^b^	OFF	Hospital 1	Hospital 2
James	OFF	Hospital 2	OFF	Hospital 2	AA	AA	OFF
Olivia	Hospital 3	OFF	OFF	OFF	Hospital 2	Hospital 3	Hospital 1
Amelia	Hospital 2	AA	AA	OFF	OFF	OFF	OFF
Joseph	OFF	Hospital 1	Hospital 3	AA	AA	OFF	OFF
Emily	AA	OFF	Hospital 1	Hospital 3	Hospital 1	OFF	OFF
Matthew	OFF	OFF	Hospital 2	Hospital 1	Hospital 3	Hospital 2	Hospital 3
Laura	OFF	OFF	OFF	AA	OFF	OFF	AA

^a^AA: Attend Anywhere.

^b^OFF: off day.

**Table 4:** Mileage for the first objective.

	October 14, 2019	October 15, 2019	October 16, 2019	October 17, 2019	October 18, 2019	October 19, 2019	October 20, 2019
Kelly	0	30	0	0	0	0	20
James	0	25	0	25	0	0	0
Olivia	30	0	0	0	0	30	20
Amelia	17	0	0	0	0	0	0
Joseph	0	0	38	0	0	0	0
Emily	0	0	32	18	32	0	0
Matthew	0	0	0	29	35	0	35
Laura	0	0	0	0	0	0	0

**Table 5:** Mileage for the second objective.

	October 14, 2019	October 15, 2019	October 16, 2019	October 17, 2019	October 18, 2019	October 19, 2019	October 20, 2019
Kelly	0	0	0	0	0	0	0
James	0	0	0	0	0	0	0
Olivia	0	0	0	0	0	0	30
Amelia	0	17	0	0	0	0	0
Joseph	0	0	0	0	0	0	0
Emily	0	0	0	0	0	0	0
Matthew	0	0	0	0	0	0	0
Laura	0	0	0	0	0	0	0

## Discussion

### Principal Findings

In this study, we demonstrated that mathematical modeling can be used to determine a staff schedule for an adolescent hybrid telemedical service. We developed a mathematical program that schedules mental health care practitioners and assigns them to either the base of the telehealth care service or to hospitals where staff would be required to assess or treat patients. Our approach was able to find a minimum coverage for staff by satisfying time- and location-dependent demands and to minimize travel distances.

### Findings and Contributions to the Literature

As demonstrated in our experimental study, our mathematical model can assign members of staff dates and locations. Although members of staff may be available during many days in the planning horizon, there is no guarantee that the solution provides a levelled assignment of staff to the locations. In Table 3, for example, the second member of staff (James) was never assigned to hospital 1 or 3, while Laura was never assigned to work on site at a hospital. In future work, a constraint that assigns a member of staff at least, for example, once in a fortnight to a hospital and working remotely, might be desirable.

### Limitations

The presented mathematical model has limitations because of its deterministic nature: it assumes that the demand is known in advance. However, the demand of staff needed for patients coming into hospitals has some level of uncertainty and cannot be estimated precisely. To create a more robust solution that considers uncertainty of demand, for example, a stochastic approach to the problem can be applied. In this scenario, we would assume that once a staff member is assigned a shift at a hospital, they cannot be relocated. Instead, an agency member is required to cover the unmet demand.

### Conclusions

In this study, we have presented an approach to use mathematical programming for scheduling staff to a hybrid telehealth service to evaluate children’s and adolescents’ mental health. Our objectives were to find a minimum staﬀing pattern to not only run the service but also minimize travel times of staff working at various locations including the base of the telehealth service. In times of the COVID-19 pandemic, this base can also be the home of the staff member. In future work, we will extend our approach to incorporate demand uncertainty in a stochastic programming approach with the aim to provide a robust staﬀing pattern. Furthermore, we will increase the granularity of the planning horizon, for example, mornings and afternoons, such that staff members might be repositioned during the day, responding to the demand in a more flexible way.

## Abbreviations

CAMHS: Child and Adolescent Mental Health Service
CWTCH: Connecting with Telehealth to Children in Hospital and Healthcare
NHS: National Health Service

## Data Availability

The data to run the mathematical model are provided, for example, in Tables 1 and 2 in the manuscript.
